# Perceptions of Psychiatric Mental Health Nurses Regarding the Needs of Family Members Caring for People Living With Mental Illness: A Qualitative Study in Lobatse, Botswana

**DOI:** 10.1111/jpm.70145

**Published:** 2026-05-15

**Authors:** Keletwaetse Sakwape, Gaotswake Patience Kovane, Precious Chibuike Chukwuere, Miriam Mmamphamo Moagi, Rorisang Machailo

**Affiliations:** ^1^ NuMIQ Research Focus Area, Faculty of Health Sciences North‐West University Potchefstroom South Africa; ^2^ Faculty of Health Sciences, School of Nursing, University of Botswana Gaborone Botswana; ^3^ Nursing Department University of Limpopo Sovenga Limpopo Province South Africa

**Keywords:** caring, family caregivers, mental health, mental illness, needs, nurses, perceptions

## Abstract

**Introduction:**

Family caregivers play a critical role in the rehabilitation and ongoing care of relatives diagnosed with mental illness. This involvement of families in the care of their relatives with mental illness not only improves patient outcomes but also encompasses the stress and burden experienced by family caregivers.

**Aim:**

This study aimed to explore and describe the perceptions of psychiatric mental health nurses regarding the needs of family members caring for people diagnosed with mental illness in Lobatse, Botswana.

**Methods:**

A qualitative explorative, descriptive, and contextual research design was used. Data were collected from 15 psychiatric mental health nurses using semi‐structured in‐depth interviews. The psychiatric mental health nurses were purposively sampled. The data were analysed using content data analysis. Comprehensive Criteria for Reporting Qualitative Research guidelines were followed.

**Results:**

The results yielded three themes: healthcare interventions, informational support, and tangible support needed by family caregivers of people living with mental illness.

**Discussion:**

Family members caring for individuals diagnosed with mental illness need various support interventions to help them cope with the strain of caregiving and alleviate the impact of caregiver role strain.

**Implication for Practice:**

Mental health nursing care that recognizes the need for caregiver‐oriented care practice is needed to promote a better quality of life and enhance caregivers' coping with caregiver role strain.

## Summary

- What is known on the subject?
  ○ Due to deinstitutionalisation, the care of patients diagnosed with mental illness (PDMI) has been shifted to family members
  ○ Literature indicates that due to the demands and responsibilities of the caregiving role, the family experiences a caregiver burden, which necessitates addressing their needs to help them cope with the burden
- What does the paper add to existing knowledge?
  ○ To our knowledge, this is the first research providing a comprehensive exploration of the perceptions of psychiatric mental health nurses regarding the needs of family members caring for PDMI in Lobatse, Botswana.
  ○ The key findings indicate that family caregivers of PDMI experience significant burden, resulting in unmet needs that require attention to support them in coping with their caregiving responsibilities. The identified needs include healthcare interventions, informational support, and tangible support.
- What are the implications for practice?
  ○ There is a need to integrate family caregivers' management with their loved ones' management.
  ○ Mental health practice should demonstrate knowledge in caring for caregivers of PDMI to help them cope with the burden of caregiving, which can be achieved through addressing their needs.

## 1 Introduction

Global trends indicate that numerous countries have moved away from institutional psychiatric care, thereby placing the responsibility of primary caregiving for individuals with mental illness on their families (Modie‐Moroka 2016; Monyaluoe et al. 2014; Urek 2021). Patients diagnosed with mental illness (PDMI) and in need of care depend on family caregivers for medication support and assistance with daily activities. The quality of care they receive at home greatly influences the progression of their illness (Alyafei et al. 2021). In addition to managing the patient's illness, family caregivers play a critical role in the rehabilitation and ongoing care of PDMI at home (Akbari et al. 2018). However, the literature indicates that involving families in the care of their mentally ill loved ones not only leads to improved patient outcomes, but the stress and burden encountered by the family caregivers are also highlighted (Ballal et al. 2019; Cham et al. 2022; Oriya and Alekozai 2022).

In India, factors such as the custom of interdependence among family members and concern for the family during difficult times influence the family caregivers' extensive involvement in the care of their loved ones diagnosed with mental illness (Chakrabarti 2016). Familial interdependence represents a cultural value and practice that emphasizes the sharing of intangible support, like companionship and nurturing, alongside tangible assistance, such as financial aid and caretaking, among family members (Toyokawa and Toyokawa 2019). For caregivers of PDMI, this family responsibility involves monitoring their mental health and being alert to unusual behavioural changes. Family caregivers are compelled to cope with the behavioural instability of the mentally ill family member, which may include social isolation, stigma, reduced social contact, loneliness, loss of honour, humiliation, fear, and heightened awareness (Dijkxhoorn et al. 2022). Similarly, family togetherness has been observed as a key cultural value in rural Chinese communities, contrary to Western nations where individualism is the norm (Peng et al. 2022). Since family members are seen as an entity and indispensable components of the family, they are characteristically expected to look out for one another. In providing care to their loved ones diagnosed with mental illness, the family members employ adaptive coping mechanisms and learn to control how to express their experiences and feelings; yet this process has a negative bearing on their personal lives (Hsiao et al. 2020; Peng et al. 2022).

Sub‐Saharan Africa is still at a developmental stage in providing support to family caregivers. Thus, it is important to provide mental health nursing services to family caregivers of PDMI in this sub‐region. The difficulties in providing care to PDMI in this region are exacerbated by inadequate community‐based infrastructure (Opondo et al. 2020; Verity et al. 2021). The economic disparities in developing countries negatively impact the quality of life of PDMI due to the shortage of effective healthcare services, worsening the caregiver burden (Ae Ngibise et al. 2015).

Botswana also faces a paradigm shift since the 1970s (Modie‐Moroka 2016). The treatment of the mentally ill has changed over time, from the medieval pre‐penal era through the penal eras, then the period of institutionalization of the mentally ill, to the current deinstitutionalized community‐based care supported by citizen involvement and collaboration (Brooks et al. 2022; Seloilwe and Thupayagale‐Tshweneagae 2013). One of the most apparent effects of Botswana's deinstitutionalization of psychiatric care is issues with community‐based mental health care, as an increasing number of families with PDMI are customarily obligated to assume the caregiving role of their relatives diagnosed with mental illness (Modie‐Moroka 2016). Indisputably, some caregivers may be unwilling or unprepared for the role. According to (Ntsayagae et al. 2019), the caregiving role has a bearing on the family caregivers' physical, psychological, and socioeconomic welfare, as well as the ability to adapt and cope with the strain of caregiving. The caregivers regularly encounter various hitches and challenging feelings in the provision of care for their mentally ill family members, including a lack of awareness of the mental condition, delicate and challenging caregiving obligations for their mentally ill loved ones, and financial difficulties (Hsiao et al. 2020; Verity et al. 2021).

Botswana's healthcare system operates hierarchically, with three referral hospitals at the top and health posts and mobile clinics at the base of the structure (Opondo et al. 2020; Sidandi et al. 2011). Among these referral hospitals, one serves as the country's sole psychiatric hospital, with 300 beds for patients. Mental health care in Botswana is governed by the 2003 Mental Health Policy, which aims to integrate mental health services into the broader healthcare framework (Opondo et al. 2020). Individuals seeking psychiatric support typically first encounter mental health services at the primary care level, primarily through interactions with psychiatric mental health nurses (PMHNs). However, the country faces a significant shortage of specialized mental health providers in these primary healthcare facilities. As a result, individuals living with mental illness (PLWMI) are often compelled to travel long distances to access mental health services at the sole psychiatric hospital or at psychiatric units attached to general hospitals, where they are mainly attended to by PMHNs (Kealeboga et al. 2023; Seloilwe and Thupayagale‐Tshweneagae 2013).

The role of PMHNs extends beyond the care of individuals living with mental illness; it also encompasses support for their families. The needs of caregivers play a significant role in the recovery trajectory of PDMI. Families' challenges when caring for their loved ones necessitate attention from PMHNs.

It's essential to address the potential mental health issues that caregivers may experience due to the burdens of caregiving. These challenges can ultimately affect the quality of care they provide to relatives diagnosed with mental illness. Thus, adopting a holistic approach to providing mental health services to PDMI and their families is vital for fostering their overall well‐being. Therefore, this study aimed to explore and describe the perceptions of psychiatric mental health nurses regarding the needs of family members caring for people diagnosed with mental illness in Lobatse, Botswana. Therefore, this study aimed to explore psychiatric mental health nurses' perceptions of the needs of family members caring for individuals with mental illness in Lobatse, Botswana.

## 2 Methods

### 2.1 Study Design

This study used an explorative, descriptive, contextual qualitative research design to explore and describe the perceptions of PMHN regarding the needs of family caregivers of PDMI in Lobatse. The study is reported following the Comprehensive Criteria for Reporting Qualitative Research (CCQR) guidelines (Sinha et al. 2024).

### 2.2 Study Setting

The study was conducted in Lobatse, utilizing five general clinics that also cater to patients diagnosed with mental illness seeking outpatient services. Situated in the Southeast District of Botswana, Lobatse is nestled in a valley surrounded by mountains, extending northward toward Gaborone and being close to the South African border. As the district's administrative centre, Lobatse features a town council and offers a diverse range of residential accommodations, from affordable to upscale options. The surrounding areas also include small to large‐scale agricultural farms. Lobatse serves as a crucial transit point for trade between Botswana and South Africa, with two nearby border posts: Pioneer Border, located 9 km from Lobatse and leading to Zeerust (South Africa) and Ramatlabama, situated 54.3 km away, which connects to Mahikeng (South Africa). Additionally, Lobatse is home to two hospitals, including the only psychiatric hospital in the country, which has a capacity of approximately 300 beds.

### 2.3 Population and Sampling

In this study, the target population comprised PMHN working in the Lobatse District Health Management Team (LDHMT) clinics, with at least 6 months experience of working as a psychiatric mental health nurse, registered with the Nursing and Midwifery Council of Botswana (NMCB). The participants should be able to communicate in either Setswana or English. The participants were selected using a purposive sampling technique, and the sample size was determined by data saturation. To mitigate the risk of bias associated with purposive sampling, the researchers provided a detailed account of the specific criteria used for participant selection, enhancing the study's transparency and credibility. The first author, who was responsible for collecting the data, maintained a reflective diary in which she documented her thoughts, feelings, and observations throughout the research process. This diary served as a valuable resource for critical reflection and was shared with the other authors who were overseeing the study, fostering collaboration and ensuring a comprehensive understanding of the research experience.

### 2.4 Ethical Considerations

Ethical approvals were obtained from the North‐West University Health Research Ethics Committee (NWU‐HREC), with ethics number NWU‐00178‐23‐A1, the Ministry of Health (MoH) in Botswana, and the LDHMT. PMHNs participating in the study were recruited through the LDHMT coordinator, who served as the gatekeeper, and appointed administrative officers as mediators. After recruitment, PMHNs interested in participating in the study were referred to an independent person for consent negotiation. An independent person was involved to ensure that participants' consent was given voluntarily and without undue influence from the project owner. This independent role safeguards participants' autonomy, allowing them to make informed decisions freely. Detailed information, including the purpose of the study and the rights of the participants, was provided to the PMHNs before they signed the consent forms. It was explained to the participants that they were free to withdraw from the study at any point during data collection and would not be coerced into continuing their participation. Furthermore, withdrawal from participation will not affect them in any way.

The researcher ensured that all participants were treated fairly and received appropriate compensation. In line with the National Health Research Ethics Council (NHREC) guidelines (2012) and the South African Health Products Regulatory Authority (SAHPRA) TIE model (2018), participants were reimbursed for their time, inconvenience, and expenses. The researcher provided lunch and refreshments as a gesture of respect. Participants were also compensated for any expenses incurred for the study, such as transportation to attend interviews or purchasing airtime for communication with the researcher. Furthermore, the researcher prioritized the participants' well‐being and took measures to protect them from any distress or mistreatment. Before the interviews, a psychologist was informed about the study, ensuring that any participant experiencing emotional difficulty could be promptly referred for support. To ensure confidentiality and anonymity, the interviews were conducted in a private office provided in each clinic, and participants were addressed with codes instead of their names.

### 2.5 Data Collection

Data were collected through semi‐structured face‐to‐face individual interviews. An interview guide with three open‐ended questions was used during data collection; a voice recorder and notebook were used to record the interview and field notes, respectively. The first two interviews were used to pilot the research questions. Permission to audio record the interviews was obtained from the participants before signing the consent form and commencement of the interview. The open‐ended questions were followed by probes based on the participants' responses. During interviews, communication techniques such as clarification, paraphrasing, reflection, etc., were used. Data were collected until data saturation, which was after the 15th interview. Reaching data saturation determined the end of data collection when no new information emerged, and redundancy had been reached (Gray 2017; Polit and Beck 2017). The participants were addressed by their names during interviews, facilitating rapport building. Participants were assured that their names would not be used during report writing, but codes would be utilized. The interviews lasted from 45 min to an hour. Data were collected from PMHNs caring for people diagnosed with mental illnesses in five public clinics in the LDHMT.

### 2.6 Data Analysis

The collected data were transcribed verbatim. Data were analysed using content analysis. The content analysis method comprises four primary steps: decontextualization, recontextualization, categorization, and compilation (Bengtsson 2016). During the decontextualization phase, transcribed data were meticulously examined, noting significant details and nuances. Their initial impressions and insights played a crucial role in shaping the subsequent stages of the analysis. In the recontextualization stage, the original text was revisited to analyse it alongside the identified meaning units. Various colours highlighted each important component, enhancing clarity and visually illustrating their interrelationships. The categorization step involved thoroughly analysing the data to identify key concepts. Themes were developed and sub‐themes to systematically organise the findings into meaningful categories. Coded materials were classified into distinct fields aligned with the study's focus, facilitating clearer data interpretation and yielding valuable insights. The final step, compilation, entailed organising the themes based on their effectiveness in addressing the research questions. This process occurred concurrently with the writing of the analysis, fostering a dynamic interplay between data interpretation and documentation. The participants' experiences were examined and their roles in caring for individuals living with mental illness (PLWMI). This exploration aimed to reveal insights derived from their caregiving experiences, emphasizing their perceptions' complexities and transformative effects.

An independent co‐coder, who signed a confidentiality agreement, was involved in the data analysis, and consensus was reached on the categories and themes. Involving a co‐coder enhanced the rigour of the findings by ensuring they aligned with a broader perspective (Coulston et al. 2025). Additionally, the co‐coder provided valuable insights that helped to address potential biases or blind spots that the primary researcher may have overlooked.

### 2.7 Measures to Ensure Trustworthiness

This study established trustworthiness by emphasizing the accuracy of the information shared by participants and affirming the meanings they assigned to their responses. To further enhance trustworthiness and rigour, the researchers utilized the epistemological standards of dependability, confirmability, authenticity, credibility, and transferability, as delineated in the framework proposed by Lincoln and Guba (Polit and Beck 2017). Credibility was bolstered through prolonged engagement with the PMHNs, allowing for the development of meaningful relationships built on trust (Connelly 2016). This rapport allowed researchers to gather rich and detailed descriptions of the PMHNS' experiences and insights, enriching the understanding of their distinct viewpoints. Additionally, the credibility of the findings was significantly boosted by a peer review process involving an experienced co‐coder who contributed to the data analysis. This collaboration enabled a comprehensive examination of the emerging themes and sub‐themes, with both the co‐coder and researcher achieving agreement on the interpretations (Thomas and Magilvy 2011). Confirmability was ensured with the involvement of an experienced co‐coder during the data analysis process (Thomas and Magilvy 2011). An audit trail was maintained to enhance dependability, documenting all activities conducted throughout the study (Connelly 2016). This detailed documentation of each research phase promotes transparency and facilitates study replication while providing insights into decision‐making processes and potential biases (Ahmed 2024). A comprehensive description of the research setting and methodologies addressed transferability, allowing readers to evaluate the findings' applicability to similar contexts and populations beyond the study (Ahmed 2024). To ensure authenticity, all interviews followed a standardized interview guide, engaging a diverse range of participants to capture various perspectives. An audit trail of the audio‐recorded interviews was also kept (Johnson and Rasulova 2017).

## 3 Results

### 3.1 Demographic Characteristics

Out of the 15 participants (*N* = 15) involved in the study, seven were female (*n* = 7), while the remaining eight were male, forming the largest group within the sample (*n* = 8). The ages of the participants ranged from 33 to 45 years, with a mean age of 38 years (SD 4.05). The minimum years of experience as a mental health nurse were 2.5 years, while the maximum was 7 years. Each participant possessed at least a post‐basic diploma in psychiatric mental health nursing and a basic general nursing qualification. The demographic characteristics of the participants are shown in Table 1 below.

**TABLE 1:** Participants' demographic profile.

Participant	Age	Gender	Years of experience as a mental health nurse
A	42	M	5
B	39	F	3
C	41	M	5
D	44	F	6
E	39	F	4
F	35	M	3
G	37	M	3
H	45	M	7
I	30	M	3
J	33	F	2.5
K	37	F	4
L	40	F	5
M	36	F	4
N	39	M	5
O	35	M	4

### 3.2 Themes and Sub‐Themes

Following the data analysis, three primary themes and their corresponding subthemes emerged from the interviews, as perceived by PMHNs. The first theme concentrated on the healthcare interventions required by family caregivers. The second theme emphasized the informational support needed by these caregivers, while the third theme underscored the importance of tangible support for family caregivers. Table 2 illustrates the themes and subthemes reflecting PMHNs' perceptions of the support necessary for family caregivers.

**TABLE 2:** Themes and sub‐themes.

Themes	Sub‐themes
Healthcare interventions needed by family caregivers	1.1 Assessment of family needs and support during follow‐up visits 1.2 Crisis intervention and debriefing 1.3 Psychological support to deal with the psychological burden 1.4 Home visits to assess and support family members
2 Informational support needed by family caregivers	2.1 Information on the causes, symptoms, and management of mental illness 2.2 Skills training on the management of mental illness 2.3 Information on admission and legal procedures
3 Tangible support needed by family caregivers	3.1 Financial support for family caregivers 3.2 Respite care to relieve family members' burden

#### 3.2.1 Theme 1: Healthcare Interventions Needed by Family Caregivers

The PMHNs highlighted the need for interventions to ease the family caregivers' burden.

##### 3.2.1.1 Assessment of Family Needs and Support During Follow‐Up Visits

The study's findings indicated the need for assessment and support of family members caring for PDMI during their patient's follow‐up visits. The quotes below support the findings.

Participant A, Male, Clinic 1: “Comprehensive assessment of the family or caregiver can help us recognise that family is a set up that forms the base for the patient's recovery. It can help to assess if they are in a better position to sustain themselves financially, that they can cope with the challenges or what the medical personnel would expect from them”.

Participant B, Female, Clinic 3: “A comprehensive assessment should be conducted. As I mentioned earlier on, these caregivers have stress. They are suffering from depression, and experience caregiver role strain. They need to be assessed so that they be helped against all these things”.

Participant D, Female, Clinic 1: “I mean, like I touched on the issue earlier on, as we realize that they are strained in that manner, we should call them so that we meet them and hear the extent of their caregiver role strain, and how we can help them or how they think they can be helped, so that they can be able to continue caring for their patient”.

Participant E, Female, Clinic 1: “That assessment needs to be conducted because I believe it will assist in the best care taking of the patient. It will assist the health care personnel to know the burden experienced by the caretaker, what they need, and the best intervention for their needs. But without that assessment, we wouldn't know”.

##### 3.2.1.2 Crisis Intervention and Debriefing

The PMHNs reported that the family caregivers need crisis intervention and debriefing in case of psychiatric emergencies, as supported by the quotes below:

Participant F, Male, Clinic 2: “I don't know if I will be putting it right. But what I'm thinking is having access for the caregivers, there should be crisis emergency response teams everywhere in the country. Because you will never know. Our patients can be unpredictable. The caregivers should know that if something happens there is nearby help”.

Participant G, Male, Clinic 5: “If there is an incident, they should have the freedom of accessing counselling on that day or immediately after the incident”.

Participant O, Male, Clinic 5: “Caregivers can become confused when dealing with an acutely ill patient or when a patient relapses. They need to receive counselling and training on handling emergencies”.

##### 3.2.1.3 Psychological Support to Deal With the Psychological Burden

The participants are of the view that family caregivers need psychological support to deal with the psychological stress. The following quotes support their views:

Participant F, Male, Clinic 2: “They really need emotional support. Caregivers need to be supported with counselling and support groups for them to manage stress and anxiety, and to avoid depression”.

Participant G, Male, Clinic 5: “The other thing I think they need counselling and regular debriefing so that they talk about their health, mental health‐wise about caring for the patient. I guess that can help address their mental health”.

Participant I, Male, Clinic 1: “Also, as mental health personnel, realize that the caregiver is experiencing the burden, they should book their counselling separately, to prevent them from now also having a mental illness such as depression or anxiety”.

Participant J, Female, Clinic 4: “Some are emotionally strained. They need emotional support. Some are strained as they have been caring for this patient alone without anyone's support”.

##### 3.2.1.4 Home Visits to Assess and Support Family Members

The PMHNs emphasized conducting home visits to assess and support the family caregivers as supported by the quotes below.

Participant A, Male, Clinic 1: “The other thing as I have already alluded that visiting the families, to check as a nurse or health professional, which will allow you to assess further on how to address the identified needs”.

Participant D, Female, Clinic 1: “When you get home, you assess for maybe some other needs that can be visible at the home environment. The needs that maybe when they assessed in the hospital may not be easily identified until we do it at home”.

Participant K, Female, Clinic 5: “When visiting patients at their homes, healthcare providers can assess the situation firsthand. They may encounter challenging patients and families struggling to manage them, allowing for the provision of care at home”.

Participant M, Female, Clinic 2: “Conducting home visits will allow the mental health professionals to assess how the caregiver and their patient are relating while at home. This will enable the health workers to understand the caregiver better when they present their problems”.

#### 3.2.2 Theme 2: Informational Support Needed by Family Caregivers

The findings of the study revealed the family caregivers' need for information on the mental conditions the patients are diagnosed with. The PMHNs reported that the family caregivers lack knowledge of various aspects of mental health and illness.

##### 3.2.2.1 Information on the Causes, Symptoms, and Management of Mental Illness

The PMHNs reported that most caregivers are uninformed about the mental illness their patient is diagnosed with. Below is what most of them said:

Participant A, Male, Clinic 1: “It is also caused by lack of understanding from the caregivers on what their illness is and its manifestation, and reasons to be brought to the hospital when it starts. So, we experience such issues where there are conflicts between the patient and family because there is a lack of understanding of the mental illness”.

Participant B, Female, Clinic 3: “My experience is that these caregivers need comprehensive mental health education. Most of them lack knowledge and insight into mental illness. They also lack knowledge on what they should do going forward regarding family members struggling with mental illness”.

Participant E, Female, Clinic 1: “They would come crying, frustrated that they do not know the patient's condition, what caused it, etc.”

Participant H, Male, Clinic 3: “In my view, this information can include the patient's condition; what's the condition, what are the signs and symptoms, and how is it managed? As I said, they need to be educated about how they should treat the patient and their attitude towards him that will not trigger the patient into a relapse”.

##### 3.2.2.2 Skills Training on the Management of Mental Illness

The PHMNs are of the view that family caregivers need training on how to manage their patients diagnosed with mental illness. Below is what they said regarding the need for skills training of the family caregivers:

Participant A, Male, Clinic 1: “So, they may be taught how to act when one is diagnosed with mental illness”.

Participant F, Male, Clinic 2: “They don't know how to handle the patient if he behaves in a certain way at home. So, they have never been trained. I think training and education are very important in these issues”.

Participant O, Male, Clinic 5: “…and teach them these basic skills such as managing a psychiatric patient during an emergency”.

##### 3.2.2.3 Information on Admission and Legal Procedures

The participants expressed the need for family caregivers to understand the legal issues in caregiving for a patient diagnosed with mental illness. The following quotes support their views:

Participant F, Male, Clinic 2: “Some people are taking care of their relatives diagnosed with mental illness, but they do not understand the legal issues surrounding the care of mentally ill patients”.

Participant J, Female, Clinic 4: “The family caregivers also need to know the correct procedure to follow when taking a patient to the hospital when they are not well. At times, they may be unsure whether to take the patient to the clinic first or directly to the psychiatric hospital”.

#### 3.2.3 Theme 3: Tangible Support Needed by Family Caregivers

The PMHNs noted the need for tangible support for the family caregivers, citing that it would lessen their caregiver burden, thereby enhancing their coping.

##### 3.2.3.1 Financial Support for Family Caregivers

The PMHNs recommended that the family caregivers should be assisted financially for provision of necessities such as food, toiletry, and bills. They further noted that the family caregivers need support to access mental health services for their relatives diagnosed with mental illness. The following quotes support their recommendations:

Participant J, Female, Clinic 4: “They need financial support because some of them are from disadvantaged families. The grant given to patients should also be extended to their caregivers”.

Participant L, Female, Clinic 2: “So, the caregivers' grievances should be given priority. This includes their financial issues, such as buying food for the family, and money to pay for transporting the patient to the clinic for their reviews”.

Participant H, Male, Clinic 3: “One other thing I believe financial support is needed because we are talking about clients who come for check‐ups most frequently, and for one to access the health facility, there is a need for transport, and this means money. It's not easy to reach the facilities. Some patients travel long distances to reach the clinics”.

##### 3.2.3.2 Respite Care to Relieve Family Members' Burden

The PMHNs are of the view that respite care is essential in relieving the family caregivers of the burden they experience. They expressed the family caregivers' need for a break, which, through the quotes below, can be achieved through respite care.

Participant F, Male, Clinic 2: “The other one is that they need a break to avoid burnout. Taking a break can work for the caregivers. During that brief period, they are without the patient, they mostly feel relieved”.

Participant K, Female, Clinic 5: “The use of respite care centres can be a good idea. Or any place where the caregivers can temporarily leave their patients while attending to their other issues”.

Participant L, Female, Clinic 2: “If possible, if there was anywhere where the caregiver wanted to go, they would leave their patient temporarily for a certain period, for example, two days, and that's how they could be assisted”.

Participant M, Male, Clinic 2: “What I can add is that, in my view, I think there is a need for respite care services, which can assist in easing the burden experienced by the caregivers”.

## 4 Discussion

The findings indicated the need for assessment and support of family members caring for PDMI during their patient's follow‐up visits. Participants reported that the assessments can allow the healthcare personnel to assess the burden experienced by the family caregivers, how they are coping, and what can be done to assist them in coping with the burden they are facing. This is supported by a study conducted in Cambodia, which indicated that mental health assessment should be prioritized for family caregivers and backed up with psychotherapy when needed (Phoeun et al. 2023). The authors stated that an opportunity should be seized to assess the family caregivers as they accompany their relatives diagnosed with mental illness to the health facilities for treatment. Similarly, and within the African context, a study conducted in South Africa by Silaule et al. (2024) suggested the importance of integrating the assessment of the burden experienced by the family caregivers of PDMI with routine clinical practice by conducting regular assessments to find out who the family caregivers are and to determine their health status.

The participants alluded that from observation during health care facility visits of PDMI, their family caregivers appear to be emotionally overwhelmed. To this effect, the PMHNs recommend that the family caregivers be provided with emotional support through counselling, which in Botswana is provided by psychologists based at the sole psychiatric hospital in the country. This results in limited access to these services. The shortage is compensated for by the services of mental health nurses, who are trained to conduct counselling, to complement the demand for counselling services needed by PLWMI and their families. In support of the above findings, a study conducted by Moudatsou et al. (2021) indicated that the care of chronic PDMI causes various challenges for family caregivers. As a result, it is not always easy to adapt and cope at a psychosocial level. Moreover, the study found that family caregivers often feel bewildered and panicked, uncertain about how to respond during a psychiatric emergency. This underscores the necessity for crisis intervention and debriefing support. According to Moudatsou et al. (2021), these challenges can be addressed through a collaborative approach between mental health professionals and family caregivers, support groups, and group psychotherapy involving family caregivers who encounter similar situations. Extending counselling services to family caregivers provides them with good conditions for their caregiving role (Arkorful et al. 2020). Additionally, family caregivers need counselling to instil positivity in them to combat worrying about their family members living with mental illness (Anokye 2018).

The results of this study emphasized the need for conducting home visits to assess and support family caregivers. During these visits, the PMHNs can comprehensively evaluate the family caregivers, including the home environment and how they interact with their patients. Literature supports the family caregivers' home visit needs (Vukeya et al. 2022). The authors emphasized that home visits are essential for building therapeutic relationships, demonstrating the healthcare team's commitment to focusing on the patient and fostering a supportive connection. Additionally, home visits may yield a sense of positive feelings that they are not alone in the caregiving journey and will strengthen the advocacy grounds for the caregivers by mental health professionals. Furthermore, home visits reduce the family caregivers' healthcare expenses and offer a model for continuous management of PDMI (Chang and Chou 2015).

According to Redubla and Cuaton (2019), there is a need to strengthen psychoeducation for family caregivers in mental health facilities. Additionally, educating the family caregivers assists them in acquiring ample awareness and insight into the psychiatric diagnosis of their relatives. Similarly, the findings of the current study indicate that family caregivers need training and education on mental health issues. They expressed that most caregivers are uninformed about the mental illness their patient is diagnosed with; meanwhile, it is imperative to understand their diagnosis. These findings are further supported by a study conducted by Vukeya et al. (2022), which emphasized the need for psychoeducation by family caregivers. Moreover, the findings of this study indicate the need for training of family caregivers on how to manage mentally ill family members. According to Dickinson et al. (2017), to uphold quality life for the family caregivers, there is a need to integrate the educational component, which is aimed at enhancing the caregivers' knowledge of the mental illness., and the therapeutic component, which enhances their well‐being.

Our study revealed the need for financial assistance of family caregivers to ease their financial burden. Amini et al. (2023) report that one of the family caregivers' needs revealed in their study is concerns about welfare. The costs surrounding mental disorders and their treatment and provision of care to the relative diagnosed with mental illness create various financial hardships for the family caregivers. Likewise, in agreement, Chen et al. (2019) study revealed that the economic pressure on family caregivers of PDMI needed to be eased through external financial resources. Botswana lacks inpatient rehabilitation centres, respite care services, and programs that financially support family members caring for PLWMI. This situation has resulted in predominantly informal caregiving, with families serving as the primary caregivers. Consequently, the responsibility for care encompassing various aspects, including financial assistance, largely rests with the families of PLWMI. As a result, a significant theme that emerged from the interviews is the critical need for financial support for these families.

Shamsaei et al. (2015) stated that family caregivers of individuals diagnosed with persistent and debilitating mental illness require substantial practical support in various forms. Consistent with the aforementioned literature, the participants in this study believe that family caregivers require assistance in accessing mental health services for their relatives diagnosed with mental illness. Furthermore, considering the proximities of their households to access health services and the lack of financial resources, caregivers solicit the government to provide free transportation or an allowance to cover transportation costs. Similarly, in a study by Iseselo et al. (2016), caregivers required financial assistance because they lived a considerable distance from the hospital and had to use public transport or taxis to accompany their patients for reviews.

The PMHNs believe respite care is essential in relieving the family caregivers of the burden they experience. In support of the above assertion, O'Neill et al. (2022) indicated that family caregivers expressed the need for respite care and breaks to cope with the burden of caring for their relatives diagnosed with mental illness. In another study, the family caregivers conveyed a need for a safe place for their relatives diagnosed with mental illness because they viewed the community as unsafe, which contributed to their stress and burden (Vukeya et al. 2022). Taking a break can bring positivity to the burden and relief to attend to missed social activities due to the demands of caregiving.

### 4.1 Strengths and Limitations of the Study

This small‐scale study restricts the generalizability of its findings and emphasizes general mental illness, signalling a need for further research on specific psychiatric disorders. Using purposive sampling to select PMHNs may introduce bias if the sample does not represent the broader population. To address this, clear inclusion and exclusion criteria were established. Nonetheless, the results explored and provided unique views of the PMHNs under study. Some caregivers experienced emotional breakdowns after the interviews and were referred to a counsellor. Qualitative research can be subject to participant bias, which may compromise data authenticity. The researcher aimed to mitigate this risk by fostering rapport and maintaining a nonjudgmental demeanour. Additionally, researcher subjectivity presents a limitation. This concern was addressed by sharing the findings with research supervisors for critical appraisal, allowing for reflection on any assumptions that could influence the study.

### 4.2 Recommendations for Further Research

This study discovered that caregiver role strain is substantial and affects the family caregivers of PDMI. The study explored the perceptions of PMHNs regarding the needs of family caregivers of PDMI in a general sense. There is still a gap in research on the needs of family caregivers for specific mental disorders such as substance use disorders, schizophrenia, etc. Mental disorders can profoundly influence family dynamics. Research can identify the unique needs and challenges experienced by caregivers of individuals with each condition. This understanding can facilitate the development of targeted support programs. Furthermore, mental illnesses may manifest differently and be perceived in various ways across cultures. By investigating how these cultural variations impact the caregiving experience, research can lead to the creation of culturally sensitive support strategies. Additionally, the prevalence and factors associated with family caregiver burden for specific mental disorders are not known in Botswana, necessitating the need to conduct quantitative research in that regard.

### 4.3 Recommendations for Mental Health Nursing Practice

The findings of this study offer critical insights into the needs of family caregivers of PLWMI as viewed by the PMHNs. A basis for mental health practice is provided to recognize the needs of caregivers of PDMI in their quest to care for their loved ones. There is a need to confront the current culture in the mental health practice of handling the family caregivers of PDMI. Practices must integrate family caregivers' management with their loved ones' management. Literature indicates that family caregivers are silent patients (Chadda 2014; Udoh et al. 2021); hence, their management should be given equal attention to their patients. Mental health nursing practice should demonstrate knowledge in caring for caregivers of PDMI and experiencing caregiver role strain. Mental health nurses should employ caregiver‐oriented practices to assess their physical, social and psychological functioning to develop interventions targeting the caregivers' health. There could be a collaboration between mental health nurses, other health professionals, family members, and the community to develop effective caregiver‐oriented practices that target caregiver role strain.

### 4.4 Recommendations for Nursing Education

The researcher advocates for the incorporation of family‐centered care principles within nursing curricula, emphasizing the critical importance of comprehending the role of families in mental health care. It is essential for nursing students to engage in experiential learning through team‐based environments, as this approach will enhance their understanding of family dynamics and improve the quality of care provided to patients.

## 5 Conclusion

The results of this study present the views of PMHN regarding the needs of family caregivers of PDMI. The study findings contributed to psychiatric mental health nurses' perceived important needs of family members caring for people living with mental illness. The findings exemplified how multifaceted the caregiver burden is, calling for appropriate interventions to assist the family caregivers in coping with the strain of caregiving. Support for family caregivers includes assessing their needs, providing crisis intervention and debriefing during psychiatric emergencies at home, and offering psychological support. The study also highlights how critical it is that mental health nurses have a thorough understanding of the challenges that family caregivers encounter. By comprehending these dynamics, nurses can create targeted interventions that not only address the needs of patients but also assist families in navigating the intricacies of mental illness. Providing the family caregivers with thorough information on their relatives' diagnosis can enable them to have a better understanding of the mental illness involved.

Furthermore, family caregivers need tangible support such as grants and allowances to ease their financial constraints, support to access mental healthcare, and respite care to relieve their burden.

## 6 Relevance Statement

This study aims to explore and describe the perceptions of PMHNs regarding the needs of family caregivers of PDMI. In their interaction with the PDMI and their family caregivers during mental health service provision, the PMHNs have direct contact with the family caregivers. In the process, they can understand family caregivers' struggle in caring for the PDMI. Examining and describing the PMHNs' views regarding the family caregivers' needs highlights the burden faced by the caregivers and the support they need to alleviate the deleterious effects brought by the caregiving role. This research provides insights that can help identify effective interventions to reduce the caregiving role strain. Additionally, the findings may contribute to developing guidelines and policies prioritizing family caregivers' well‐being, ultimately improving the quality of care provided to the PDMI.

## Author Contributions

K.S., drafted the manuscript. G.P.K., M.M.M., P.C.C., and R.M. reviewed the manuscript. All authors authorized the publishing of the manuscript in the journal.

## Funding

The authors have nothing to report.

## Ethics Statement

The study was approved by the North‐West University Health Research Ethics Committee (NWU‐HREC), with ethics approval number: NWU‐00178‐23‐A1, the Ministry of Health in Botswana, and the Lobatse District Management Team. This study only used human participants; no animals were included. All study processes for research on human subjects, followed the 1975 Helsinki Declaration as revised in 2013, and the institutional and national health research ethics committees in charge of human experimentation ethical standards.

## Consent

Informed consent was obtained from all the study participants.

## Conflicts of Interest

The authors declare no conflicts of interest.

## Data Availability

The data that support the findings of this study are available on request from the corresponding author. The data are not publicly available due to privacy or ethical restrictions.
